# Improving the Sequence Ontology terminology for genomic variant annotation

**DOI:** 10.1186/s13326-015-0030-4

**Published:** 2015-07-31

**Authors:** Fiona Cunningham, Barry Moore, Nicole Ruiz-Schultz, Graham RS Ritchie, Karen Eilbeck

**Affiliations:** European Molecular Biology Laboratory, European Bioinformatics Institute, Wellcome Trust Genome Campus, Hinxton, Cambridge CB10 1SD UK; Department of Human Genetics, University of Utah, Salt Lake City, UT USA; Department of Biomedical Informatics, University of Utah, Salt Lake City, UT USA; Wellcome Trust Sanger Institute, Hinxton, Cambridge UK

## Abstract

**Background:**

The Genome Variant Format (GVF) uses the Sequence Ontology (SO) to enable detailed annotation of sequence variation. The annotation includes SO terms for the type of sequence alteration, the genomic features that are changed and the effect of the alteration. The SO maintains and updates the specification and provides the underlying ontologicial structure.

**Methods:**

A requirements analysis was undertaken to gather terms missing in the SO release at the time, but needed to adequately describe the effects of sequence alteration on a set of variant genomic annotations. We have extended and remodeled the SO to include and define all terms that describe the effect of variation upon reference genomic features in the Ensembl variation databases.

**Results:**

The new terminology was used to annotate the human reference genome with a set of variants from both COSMIC and dbSNP. A GVF file containing 170,853 sequence alterations was generated using the SO terminology to annotate the kinds of alteration, the effect of the alteration and the reference feature changed. There are four kinds of alteration and 24 kinds of effect seen in this dataset. (Ensembl Variation annotates 34 different SO consequence terms: http://www.ensembl.org/info/docs/variation/predicted_data.html).

**Conclusions:**

We explain the updates to the Sequence Ontology to describe the effect of variation on existing reference features. We have provided a set of annotations using this terminology, and the well defined GVF specification. We have also provided a provisional exploration of this large annotation dataset.

## Findings

### Background

The Sequence Ontology (SO) [1] provides terminology to define sequence features. These features are the building blocks of sequence annotation, and allow biologically meaningful regions to be assigned between coordinates of sequences such as genome assemblies and transcripts. The relationships between the terms in SO provide for the annotation of multi-part features such as gene models, composed of multiple transcripts, exons, introns and UTR features. Reference genome annotations are often shared using a flat file format GFF3, developed by the GMOD community [2], which stipulates that SO terms describe each annotated feature, thus many genome annotation tools use SO to describe reference genome features. While terms to describe variants have long been part of the Sequence Ontology, increased need for new variation terms to describe the predicted effect of sequence alterations on existing genomic features lead to the development of new terms. This has been driven by the proliferation of software tools that predict the effect of sequence alterations such as Ensembl’s Variant Effect Predictor (VEP) [3] and the VAAST suite tool: Variant Annotation Tool (VAT) [4]. In this mansucript, SO terms are italicized and written without underscores.

Next generation sequencing (NGS) technologies have provided an enormous expansion in our understanding of the landscape of genetic variation [5, 6] as well as the impact of that variation on human health [7–9]. These datasets create a significant burden in computational analysis and data storage, but established work-flows for analysis are emerging [3] and well established data formats exist for each stage of the process. The original base calls from the sequencer are converted to FASTQ files [10] that contain the sequence data; the SAM format [11] captures the alignment of the sequence to a reference genome and the Variant Call Format [12] has become widely adopted by variant calling tools to report variants and the information needed to call them. However, knowing the type and genomic location of a sequence change is just the first step in understanding its clinical or biological consequences. Variant annotation then begins the process of adding additional knowledge about the structural and functional consequences of those variants through the impact on reference sequence features and ultimately on phenotype.

The Genome Variation Format (GVF) [13] is a variant file format for the detailed annotation of genetic variation. GVF is a community supported format that uses established ontologies such as the Sequence Ontology [1] to describe the variant data. GVF does not replace existing variant nomenclature systems such as HGVS [14] and ISCN [15] that provide effective ways to unambiguously describe individual variants in the literature. GVF provides the infrastructure to support inclusion of these nomenclatures along with other detailed variant annotations in a format capable of supporting genome scale variant data. GVF is used in the community for exchange of variant annotations between Ensembl [16], DGVa and dbVar [17] and is compatible with existing GFF3 software [2, 18] as well as emerging domain specific tools [4, 19].

### User requirements and ontology development

**Fig. 1:**
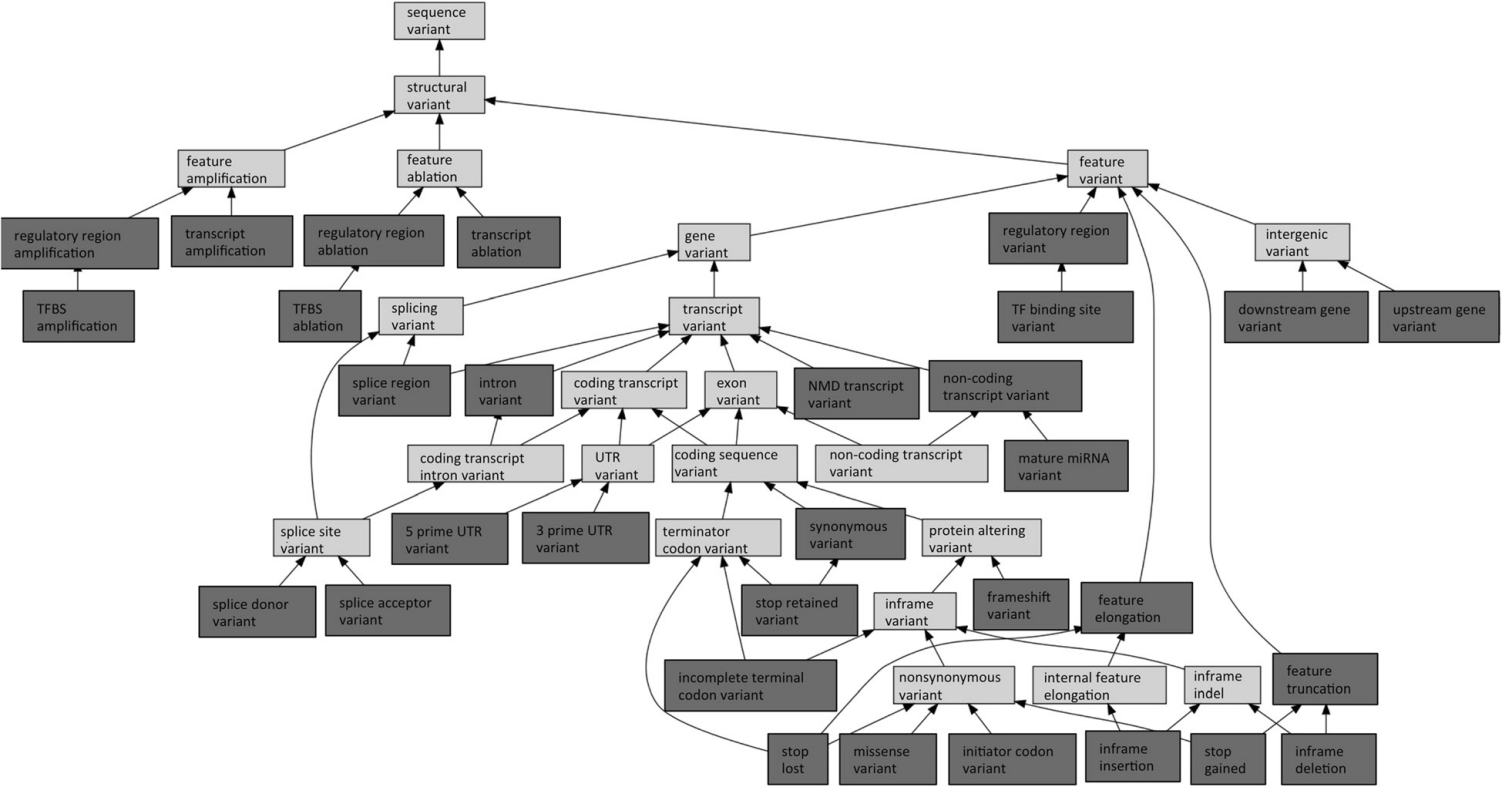
Hierarchical view of new and modified Sequence Ontology terms used by Ensembl to annotate the effects od sequence alteration. A portion of the SO *sequence variant* subsumption hierarchy is shown, with terms used by Ensembl in dark grey. *Feature variant* terms define cases where the sequence alteration occurs within or overlaps an annotated reference feature such as a transcript or exon, whereas the kinds of *feature ablation*, *feature amplification*, define cases where an entire feature is altered. Definitions for these terms are available from the miSO browser: http://sequenceontology.org/browser/obob.cgi and http://ensembl.org/info/genome/variation/predicted_data.html

Upon the release of the specification for variant genome annotation, GVF used terms from the Sequence Ontology release 2.4.3. While this resource provided 101 terms to describe the effects of a sequence alteration on genomic features, it was still missing sufficiently specialized terms to fully capture the kinds of variation annotated by the Ensembl variation pipeline [20]. A requirements analysis was undertaken to establish the terminology and relationships between terms to accomplish annotation and facilitate queries of annotated datasets. Ensembl uses 34 terms [21] to describe the effect of variation, 21 of which were new to SO, and 2 required an ammendement to the name. Figure 1 shows a subset of the terms in SO that describe sequence variants, with the Ensembl terms highlighted.

In the SO, the sequence alteration and the effects of the alteration are separated. A *sequence alteration* defines the nucleotide change observed in an individual sequence, in relation to a reference sequence. Examples of alterations are *insertion*, *deletion*, *substitution* and *SNV*. The effect of a *sequence alteration* is the observed or predicted change to annotated reference seqeunce features. These effects of sequence alterations are defined as *sequence variants* in SO and are outlined in Fig. 1. Examples of these terms are *missense variant*, whereby *codon* bases are modified in such a way as the resuling amino acid would change, and *splice donor variant* where by the alteration changes the two-base pair region at the 5′ end of an *intron*.

One of the advantages of using an ontology for the annotation of data, is that given the related nature of the terms, there are options to annotate data to the level of detail afforded by the evidence. Under the *sequence variant* node, SO provides two high level nodes in the ontology: *structural variant* and *functional variant*. Structural variants pertain to changes with regard to annotated sequence features, and are the output of automated variant effect predition tools such as VEP [3]. Functional variants however describe the cellular effect of a sequence alteration and are generally manually curated. These functional terms have largely been absorbed into the Variation ontology [22] and are not automatically assigned by variant effect prediction tools. With regards to *structural variants*, the alteration can either internally modify a sequence feature, when the alteration falls within the extent of a reference sequence feature such as an exon *(feature variant*), or the alteration can be greater than the extent of the sequence feature, causing the ablation or amplification of an entire genomic feature such as a transcript.

The *feature variant* node in the ontology subsumes the terms that describe changes internal to genomic features such as those affecting genes, transcripts and introns. The majority of the sequence alterations currently annotated by Ensembl cause *feature variants*. These feature variant terms are shown in Fig. 1, where the terms used in Ensembl annotations are highlighted in dark grey. There are five subtypes: *intergenic variant*, *gene variant, feature truncation, feature elongation* and *regulatory region variant*. Of these terms, *gene variant* has 77 direct and indirect subtypes and includes most of the terms that describe structural sequence variants caused by substitutions and small insertions and deletions. This portion of the SO contains terms with multiple parents, to allow for effective querying of the annotations. For example, the term *stop retained variant* is both a *synonymous variant* and a *terminator codon variant*. Users are thus able to query the Ensembl data for all terminator codon variants or all synonymous variants.

### Annotated variants

**Fig. 2:**
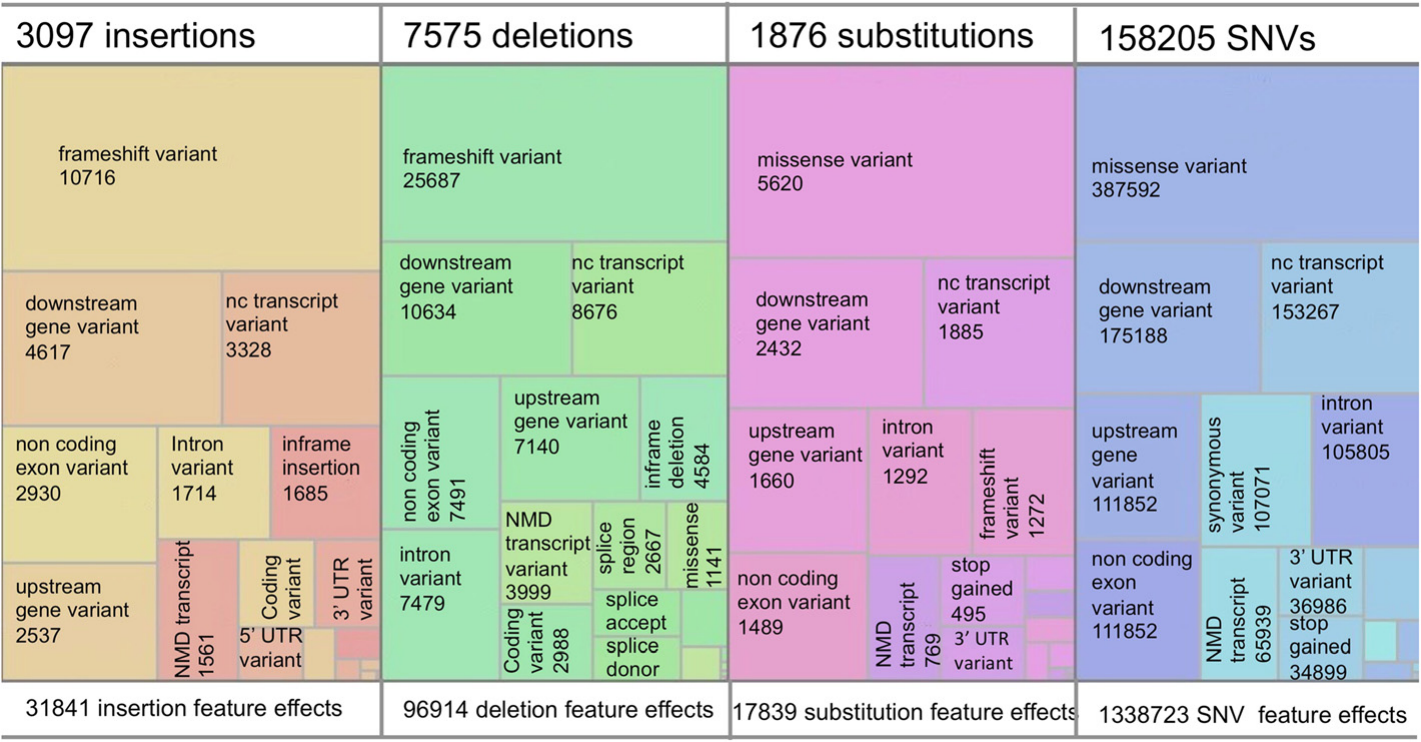
Treemap of the proportion of variant affect atributed to each kind of sequence alteration in Ensembl human GVF dataset (release 69). A treemap displays hierarchcal data as nested rectangles. In this dataset there are four kinds of sequence alteration annotated: *insertion*, *deletion*, *substitution* and *SNV*, each with a different color. For each sequence alteration, the annotated variant effects are shown with the size of the rectangle proportional to the number of occrurences of that annotation, and the count is provided where space permits. The treemap was generated using the IBM Manyeyes tool (http://www-958.ibm.com/)

GVF formated variant genome annotations for 19 organisms, typed using SO are available within the Ensembl databases [23] and for download (ftp://ftp.ensembl.org/pub/release-69/variation/gvf/). Included in this set is a GVF file of 170,853 human variant annotations, with data from dbSNP [24] and COSMIC [25] using the described terminology. There are four kinds of sequence alterations reported, corresponding to 158205 *SNVs*, 7575 *deletions*, 3097 *insertions* and 1876 *substitutions*. There are 24 kinds of variant_effect reported in the file, and five kinds of genomic feature affected (*mRNA*, *miRNA*, *transcript*, *primary_transcript* and *ncRNA*). There are 1,485,317 reported variant effects with corresponding genomic features, as a single alteration may perturb many annotated genomic features. For example an SNV may intersect two alternate transcripts, one in an exon, the other in an intron. Figure 2 shows a tree map of the proportion of variant effects annotated to each kind of sequence alteration in this dataset. As can be seen, each kind of alteration causes proportionally different effects upon the genome features; insertions and deletions cause more frameshift variants, where as the SNVs and other substitutions cause more missense variants.

## Discussion and conclusions

Detailed annotation of sequence variation is complicated because reference genome annotations are complex. Genes may produce multiple transcripts, may overlap each other on opposite strands, or even be nested within introns of other genes, therefore a variant may influence multiple genomic features. Capturing the effect of a sequence alteration on the genomic features with which it intersects is an important step towards understanding the implication of the variant sequence. The terminology described here provides a basis with whch to categorize and define sequence variation and the flexibility to annotate the effect with respect to the feature intersected. This ontology provides very specific leaf terms, with which to automatically annotate genomic sequence but also useful mid level terms for querying.

Future developments to the ontology will include developing relationships between the sequence variant terms and the sequence features that are affected. There has been significant uptake of these variant effect terms by the genomic variant annotation community. The UCSC genomic browser uses this termnology in variant annotation [26] as does the NCBI’s ClinVar data dictionary and dbVar database [17]. New terms will be added as required. New terms and updates to the ontology may be requested using the term tracker (https://sourceforge.net/p/song/term-tracker/). Development of the SO is collaborative, incorporating community discussion via our mailing list and the term tracker as well as the results of focused working groups.
